# Spatial memory-based behaviors for locating sources of odor plumes

**DOI:** 10.1186/s40462-015-0037-6

**Published:** 2015-05-04

**Authors:** Daniel Grünbaum, Mark A Willis

**Affiliations:** School of Oceanography, University of Washington, Seattle, 98195-7940 WA USA; Department of Biology, Case Western Reserve University, Cleveland, 44106 OH USA

**Keywords:** Bayesian estimation, Gaussian puff model, odor source location, moth behavior, *Manduca sexta*

## Abstract

**Background:**

Many animals must locate odorant point sources during key behaviors such as reproduction, foraging and habitat selection. Cues from such sources are typically distributed as air- or water-borne chemical plumes, characterized by high intermittency due to environmental turbulence and episodically rapid changes in position and orientation during wind or current shifts. Well-known examples of such behaviors include male moths, which have physiological and behavioral specializations for locating the sources of pheromone plumes emitted by females. Male moths and many other plume-following organisms exhibit “counter-turning” behavior, in which they execute a pre-planned sequence of cross-stream movements spanning all or part of an odorant plume, combined with upstream movements towards the source. Despite its ubiquity and ecological importance, theoretical investigation of counter-turning has so far been limited to a small subset of plausible behavioral algorithms based largely on classical biased random walk gradient-climbing or oscillator models.

**Results:**

We derive a model of plume-tracking behavior that assumes a simple spatially-explicit memory of previous encounters with odorant, an explicit statistical model of uncertainty about the plume’s position and extent, and the ability to improve estimates of plume characteristics over sequential encounters using Bayesian updating. The model implements spatial memory and effective cognitive strategies with minimal neural processing. We show that laboratory flight tracks of *Manduca sexta* moths are consistent with predictions of our spatial memory-based model. We assess plume-following performance of the spatial memory-based algorithm in terms of success and efficiency metrics, and in the context of “contests” in which the winner is the first among multiple simulated moths to locate the source.

**Conclusions:**

Even rudimentary spatial memory can greatly enhance plume-following. In particular, spatial memory can maintain source-seeking success even when plumes are so intermittent that no pheromone is detected in most cross-wind transits. Performance metrics reflect trade-offs between “risk-averse” strategies (wide cross-wind movements, slow upwind advances) that reliably but slowly locate odor sources, and “risk-tolerant” strategies (narrow cross-wind movements, fast upwind advances) that often fail to locate a source but are fast when successful. Success in contests of risk-averse vs. risk-tolerant behaviors varies strongly with the number of competitors, suggesting empirically testable predictions for diverse plume-following taxa. More generally, spatial memory-based models provide tractable, explicit theoretical linkages between sensory biomechanics, neurophysiology and behavior, and ecological and evolutionary dynamics operating at much larger spatio-temporal scales.

**Electronic supplementary material:**

The online version of this article (doi:10.1186/s40462-015-0037-6) contains supplementary material, which is available to authorized users.

## Background

The problem of locating sources of odorant plumes is a key component of foraging and reproductive behaviors in diverse taxa [1-4]. Among the most impressive and best-studied examples of these behaviors are reproductive behaviors of male moths, such as *Manduca sexta*, that are often able to locate pheromone-emitting females rapidly even when they are distant and visually cryptic [5,6]. *M. sexta* males are capable of this behavior due to specializations in both physiology and behavior. Physiological specializations include, for example, olfactory systems that can detect and identify highly diluted mixtures of pheromone and air molecules, and that appear to be specialized to encode the complex structure of turbulent plumes [7-13]. Behavioral specializations include movement strategies such as “counter-turning” and “casting”, which are currently thought to be pre-programmed in the central nervous system of plume tracking insects [14,15]. In counter-turning, moths make systematic, stereotyped lateral (cross-wind) excursions in the presence of odorant so that their trajectories repeatedly oscillate across the plume as they progress upwind. In casting, most species of moths studied to date respond to loss of chemosensory signals by widening their lateral movements and halting or reversing upwind advances, in an effort to relocate the plume [16-18]. This is thought to function as a mechanism to relocate the plume. In one instance male gypsy moths, *Lymantria dispar*, were observed to re-contact the odor plume closer to the source than where they lost it. This unusual behavior could be explained by a relatively slow shift in wind direction coincident with the rapid re-orientation of the moth to the new wind direction [19].

Adroit alternation between counter-turning and casting appears to be central to the moths’ effectiveness at odor source location. Because they are prominent elements of the biology of most moth species studied to date [5], and they are analogous to important behaviors in many other taxa [3,4,20], the mechanisms underlying the generation of these looping zigzagging flight tracks have long been a subject of study [21-25]. However, important physiological and behavioral elements of odorant source location – in particular, which signal processing pathways are present, how their outputs translate into movement decisions, and how realized movement decisions compare in various performance metrics to hypothetical alternatives – remain poorly understood.

The physical properties of odorant plumes under typical atmospheric conditions make location of female moths and other odorant sources difficult. For example, a field study using male *Plodia interpunctella* moths showed that the maximum downwind detection distance of plumes released from arrays of artificial pheromone sources scaled with the square root of the number of sources, from roughly 15 *m* for a 2×2 array to roughly 75 *m* for a 10×10 array [26]. Their meta-analysis of pheromone trap data, after excluding extremely high saturating pheromone emission rates, found a similar square root dependence across a diversity of insect taxa. These observations demonstrate that moths’ source location success rates are strongly limited by the concentration and spatial structure of pheromone plumes under natural conditions, a conclusion that has also emerged from laboratory studies [23,27,28].

The critical challenge in odor source location imposed by typical real-world atmospheric conditions is that an odorant plume, which would have a coherent, relatively smooth distribution in steady laminar flow, is disrupted and spread by atmospheric turbulence and shifting winds. Plumes in steady, laminar flows (and in many laboratory flight tunnels) are typically narrow and continuous, presenting a clear and largely uninterrupted “trail” that can be tracked directly to the source. In general, under steady laminar flow conditions even simple searching behaviors are often sufficient to quickly and reliably locate odorant sources. For example, recent studies of blue crabs, *Callenectes sapidus*, tracking plumes in laminar flow tanks have shown lateral steering controlled by differences in odor concentration detected by chemosensors arrayed near the tips of the legs, together with upcurrent walking speed controlled independently by odor encounter rate detected by the antennules, reliably delivers the crabs to a food source [29,30].

However, most plume-following in nature occurs under unsteady conditions in which the direction and speed of the wind, and thus odorant molecule transport, is constantly shifting. Furthermore, the flow regime is typically complicated by turbulent eddies over a wide range of length scales, and is frequently obstructed by vegetation or other obstacles, so that odorant is widely dispersed and local odorant concentration is irregular and highly unpredictable. As a result, the odor concentrations encountered by a moth during any single transit through a plume in turbulent flow may be a highly misleading indicator of overall plume geometry.

Previous studies suggest strong selection for maximally effective use of the information encoded in the spatial patterns of pheromone detection [31,32]. It is clear that male moths detect and alter behavior in response to odor plume structure, and that their tracking performance differs in plumes of different chemical composition and spatial/temporal structure [24,27,28,33,34]. Because of the inherently faster speed of flight, moths encounter odor plumes at a much higher rate than walking plume trackers like crabs, and their behavioral control systems appear to be adapted accordingly. Perhaps because moths are moving through odor fields too rapidly for the brain to process the input, the steering of their counterturning track is thought to be pre-programmed, and is triggered and maintained by encounters with odor filaments in the plume [1]. In a manner similar to blue crabs, moths are thought to modulate their upwind steering according to characteristics of the plume’s structure such as odor encounter rate [24,33]. It is currently unknown to what extent turning might be informed by asymmetric stimulation of the two antennae. In both of the above examples cues indicating direction to the source are indirectly provided by the wind direction [1,2].

The apparent selective importance of rapid, accurate odor source location creates an expectation that moth behaviors are highly evolved and may, within biological constraints, approach nearly optimal performance. This expectation has motivated a number of simulation studies aimed at understanding the basic biological requirements for effective plume-following behaviors, and the specific algorithms adopted by mate-seeking moths [35-39]. It has also inspired a number of robotics studies and other engineering applications of odor source location algorithms, many of them either explicitly biomimetic or incorporating behavioral elements previously observed in moths and other animals [40,41].

In previous models of moth behavior, responses to pheromone have been assumed to belong to one of two classes: (*i*) endogenous patterns such as oscillations with frequencies that are fixed or modulated according to plume structure, or (*ii*) time-dependent responses at the receptor or neuron level that process encounters with detectable levels of pheromone [14,22,42,43]. Previously proposed behavioral algorithms for plume-tracking that are based on these instantaneous responses to odorant detection typically have low success rates under challenging atmospheric conditions, compared to success rates observed in real moths [35,36]. This suggests that key elements of moths’ plume-following behaviors may be absent in these simulation studies.

In this paper, we pose the question, what would plume-following behavior look like if moths made the “best possible” use of spatial and temporal information contained in their encounters with odorant plumes? Consideration of the information-gathering problem faced by the moths, together with analysis of real moth behavior in the laboratory and field, suggest to us that a spatial memory encoding times and positions of previous encounters with olfactory signals, coupled with simple algorithms exploiting this memory to estimate their source location, would both greatly increase the effectiveness of proposed plume-following behaviors and also parsimoniously explain some observed features of moth trajectories. Although spatial memory is well known to underly insect orientation and navigation [44], it has never been proposed, or been tested, to function in the context of odor plume tracking.

While concentration in turbulent plumes is highly unpredictable in an instantaneous sense, it is relatively much more predictable in a longer term sense that “averages” over many turbulent eddies and filaments [45,46]. Consequently, behavioral algorithms that accumulate information over a sequence of encounters with a plume should in principle be more effective at locating its source than related behaviors that discard information contained in previous encounters. In previous investigations, it has been assumed that flying male moths move through the plume too fast to sense average odorant concentration values that would provide information useful for steering [45]. Thus, neither the biological basis of such memory-based steering algorithms nor the mathematical formulations that would allow them to be systematically studied have been sufficiently investigated in the literature to establish whether behaviors with spatial memory are present in plume-tracking animals, or whether they are in fact more effective than alternative types of odor source location behaviors.

Here, we derive plume-tracking algorithms inspired by observations of male *M. sexta* moths that incorporate spatial memory and movement decision-making based on accumulated information as central elements of odorant source location behaviors. More specifically, we hypothesize that moths possess a limited short-term memory of the lateral positions of previous encounters with pheromone in absolute “geostationary” coordinates (i.e., in fixed coordinates that do not change when the moth moves or the plume shifts). We use simulations to show that this resampling over recurrent plume encounters is a potentially effective way both to improve estimates of turbulent plume geometries and also to detect changes in geometry caused by wind shifts. Thus, the availability of a spatial memory, even in a simplified form, can substantially increase the speed and accuracy of odorant source location. From a statistical perspective, this type of memory represents one approach towards understanding “best use” of spatial patterns in pheromone plumes.

To implement our model, we conceive of moths’ plume-following behaviors as composed of two interacting behavioral modules: a module responsible for spatial memory of encounters with pheromone and for deriving estimates about possible plume locations and intensities relative to instantaneous wind direction, and a separate module responsible for movement decisions based on current source location estimates. In this framing of the odor source location problem, we focus exclusively on how moths utilize spatial data, without considering in detail specify sensory processes (vision, inertial navigation, etc.) by which spatial data are obtained. These modules reflect bi-directional feedbacks we hypothesize to be present in moth behaviors: the spatial memory provides the basis for movement decisions, and movement decisions determine the subsequent encounters with pheromone that augment spatial memory.

Our analysis approaches odor source location essentially as a cognitive process deriving strategic decisions from spatial memory. A concern about this approach is whether it is plausible that moths are capable of the information processing and retention required to acquire and exploit a functional spatial memory. To gain perspective on what specific capabilities are necessary, we develop a mathematical formulation for the estimation module based on Bayesian statistical theory via conjugate priors [47]. In our model, all spatial and temporal elements of memory are encoded as a small number of Bayesian conjugate prior hyperparameters. Moths’ estimates of plume geometry are updated upon new encounters with pheromone by simple arithmetic operations. This approach endows simulated moths with the capacity to integrate information about when and where odorant was detected over iterated encounters with the plume, with surprisingly modest demands on memory and information processing. We argue that the feasibility – with minimal neural processing – of effective cognitive spatial strategies for odor source location make these and related algorithms high priorities for future experimental and theoretical investigation.

We also implement some simplified examples of the movement decision module. In our simulations, this module determines the inter-related geometrical characteristics of transits across the plume, such as flight and track angles, time delays between turns, lateral extent and up/down-wind excursions of transits, etc. We defer to another paper a serious attempt to assess which specific algorithms and parameter values best reflect behaviors used by male moths. Instead, here we take a heuristic approach, using pre-existing laboratory observations of plume-seeking movements to look for previously undetected statistical patterns in moth plume-tracking behavior. Spatial memory-based behavioral hypotheses suggest testable predictions for patterns of variation in transit geometries of *M. sexta* males. We show below that some of these predicted patterns are consistent with observed moth trajectories. We simulate behavioral modules inspired by these patterns and show that, in combination with spatial memory encoded by the Bayesian estimation scheme, they constitute relatively successful odor source location algorithms under challenging conditions of sparse chemical signals and strong atmospheric disturbances.

Finally, we use several metrics of performance to conduct an initial exploration of how odor source location success varies as a function of behavioral parameters. In nature, odor source location for male moths is typically “successful” for a moth that traces the plume to the female before she stops emitting pheromone and before she is found by another male. Thus, male moths must avoid both of two distinct modes of failure: They must minimize losses of contact with the plume; such losses are reduced by behaviors in which the moths make small upwind excursions with large lateral extents. They must also minimize the probability of arriving behind a competitor, by advancing towards the odor source as quickly as possible; rapid advances are facilitated by behaviors in which moths undertake large upwind excursions with small lateral extents. We find that performance metrics reflecting these two modes of failure are enhanced by different behavioral traits. Hence, no single set of behavioral parameters excels at all of our performance metrics, suggesting female-seeking male moths and other animals locating the sources of odor plume are likely to adjust their behaviors to cope with complex, context-dependent performance tradeoffs.

## Results and discussion

### Analysis of observed moth trajectories in the context of spatial memory

To inform our behavioral models, we reanalyzed a set of movement trajectories of male *M. sexta* moths seeking the source of an artificial pheromone plume in a laboratory wind tunnel (see experimental details in [48]). Each of 19 individuals was observed four times during a single experimental day, and the experiment was conducted over several days. Thus, 76 plume tracking flight trajectories were analyzed with a total of 458 cross-plume transits (i.e., excursions). Analysis of these trajectories with a one-way repeated measures analysis of variance showed that there were no statistically significant differences in any movement parameter (e.g., air speed, ground speed, course angle, track angle, etc.) across experimental treatments, no evidence of systematic differences across successive trials, and no indications of learned responses to experimental conditions. The moth movement data are 3-dimensional; however, data analyses presented here reflect 2-dimensional projections onto the horizontal plane for ease of comparison with our simulation results. Relationships between vertical and horizontal movements are described elsewhere [49].

**Figure 1:**
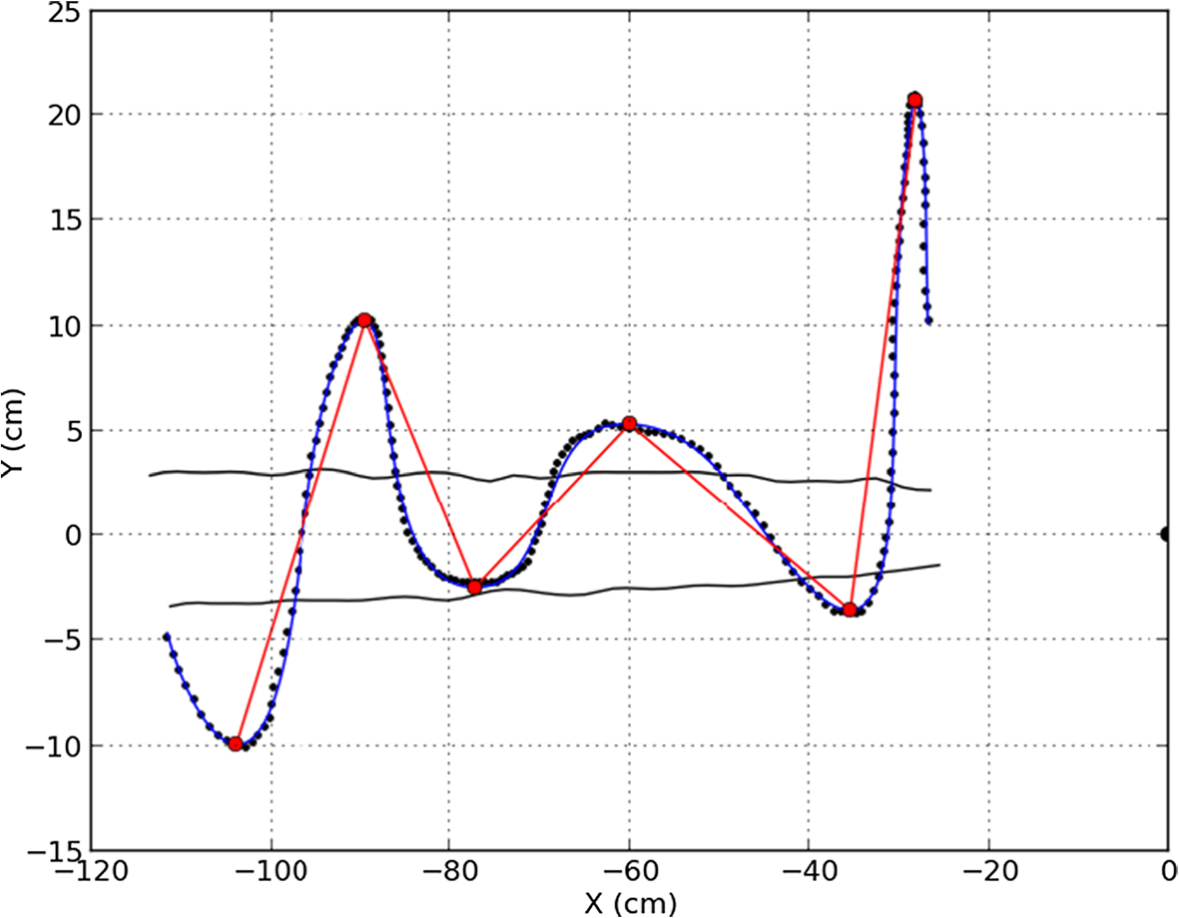
Typical trajectory of a male *Manduca sexta* moth in an experimental wind tunnel. Wind direction is from right to left (in the negative *x*-direction) at 100 *c* *m*/*s*; moth flight direction is from left to right (in the positive *x*-direction). Small black circles indicate the raw path digitized from sequences of video frames, at 30 frames per second (see [49] for other experimental details). The blue line represents a corrected path after filtering to remove frame-rate noise. The red dots indicate maximum lateral excursions. The analyses in Figure 3 are based on straight-line connections between these excursion endpoints, shown here as red line segments. The semicircle at right is the pheromone source position. Black lines indicate the approximate edges of the time-averaged pheromone plume, as determined by titanium oxide smoke visualizations and electroantennogram assays.

**Figure 2:**
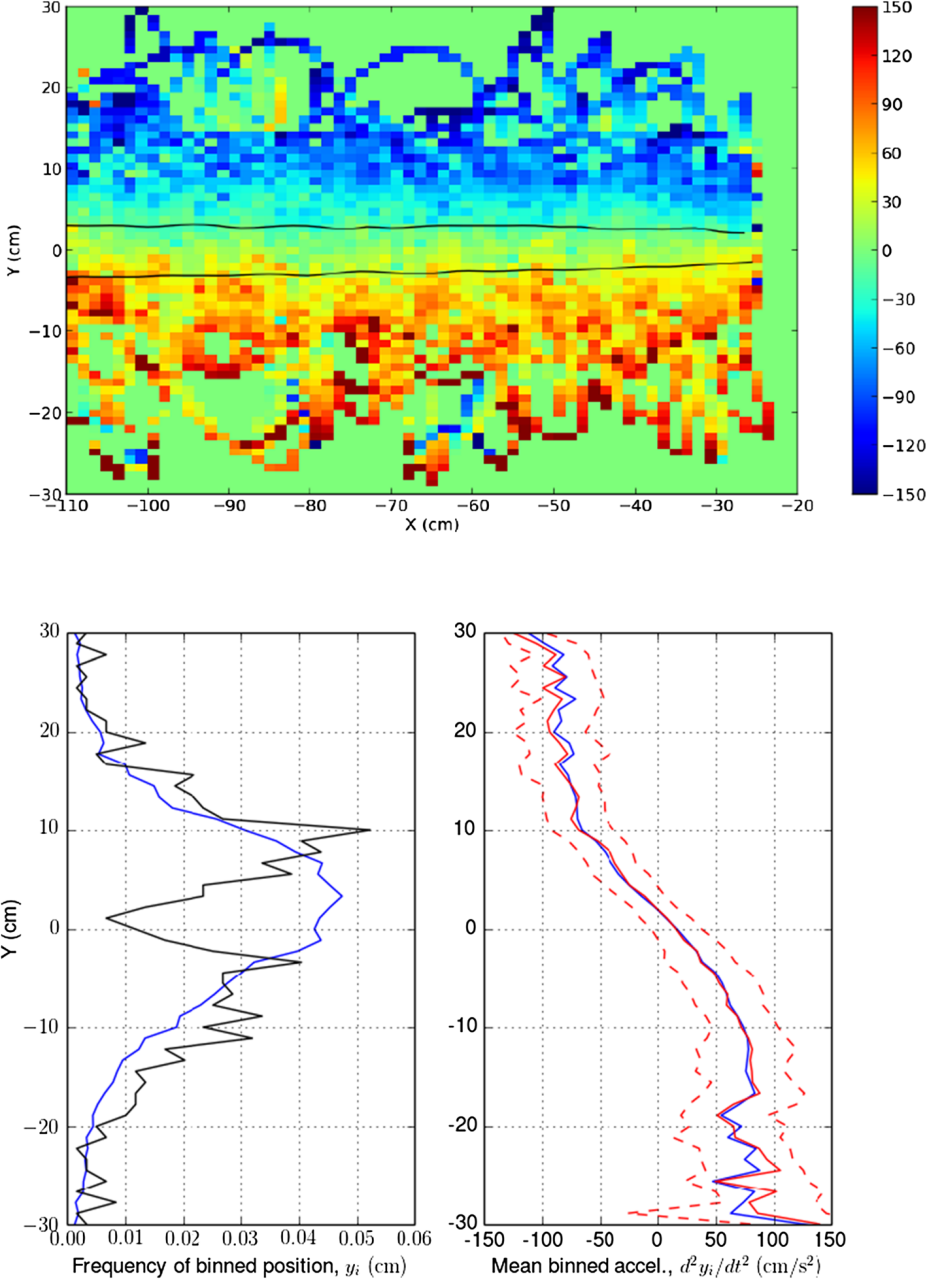
Positions and lateral accelerations observed in male moths during location of the source of a laboratory pheromone plume. Data are from 19 moths, with four digitized trajectories per moth. (Top) Average lateral acceleration of moths as a function of binned position, indicated with a color map (blue: −150 cm/*s* ^2^, red: 150 cm/*s* ^2^). Bins with no data are plotted with value zero (green). (Bottom left) *x*-averaged probability density functions (pdfs) of lateral (*y*-direction) position relative to the plume source (blue line), and of excursion endpoints (black line). (Bottom right) *x*-averaged lateral acceleration plotted as a function of binned lateral position (blue line). Also shown are medians (solid red lines) and 25*th* and 75*th* percentiles (dashed red lines) of binned data. Note that moths’ lateral acceleration towards the plume centerline typically begins before they exit the time-averaged plume, suggesting that the decision to turn has already been made or anticipated.

Despite some important differences between laboratory plumes and natural mate-seeking conditions, males exhibited characteristic counter-turning flights (Figure 1). Under experimental conditions, male moths’ cross-wind transits typically lasted approximately 0.5*s* [48]. Figure 2 shows mean lateral acceleration and the lateral extent of transits as a function of position relative to the plume, averaged over all trajectories of all observed moths. While the ensemble includes some transits with exceptionally wide or narrow lateral excursions, most transits ended relatively close to the point of departure from the time-averaged plume. Also, in most transits, lateral accelerations towards the plume centerline initiated very close to or inside the time-averaged plume boundary. These statistical features suggest that moths may be relatively accurate in estimating time-averaged plume geometry.

If so, this accuracy is remarkable in light of other data, which suggest the instantaneous pheromone distribution is so sparse and variable that information from any given transit poorly constrains the time-averaged plume geometry. These data, obtained in a different study but with the same experimental configuration and protocols, were obtained using electroantennograms, in which electrodes monitor neural responses to pheromone detected by antennae surgically removed from male moths [26,49]. Under typical experimental conditions, those measurements showed that males encountered detectable pheromone “puffs” – localized pheromone concentrations sufficient to trigger discrete, above-threshold neural responses – at an average rate of approximately 2 Hz [48]. Hence, under experimental conditions, moths likely encountered on average approximately one puff per cross-plume transit.

If we assume that pheromone puffs are encountered randomly (as Poisson points) this average per-transit encounter rate (*ρ*_*puff*_≈1) suggests that slightly more than one in three transits results in no puff detections. A comparable fraction of transits result in one puff detection, and only slightly more than one in four transits results in two or more puff detections (corresponding to *k*=0, *k*=1, and *k*≥2 in Equation 15; see Section “Estimates of puff density within the plume” for additional details, and Tables 1 and 2 for mathematical symbols and their interpretations). Under field conditions, with larger-scale 3-dimensional turbulent eddies and gusts, the frequency of transits with few or no pheromone puff encounters is likely to be even higher. These probabilities suggest a potential mismatch between the apparent accuracy with which moths estimate pheromone plumes’ time-averaged puff distributions, and the small sample size of puffs available to perform this estimate during any single cross-plume transit.

**Table 1:** **Summary of symbols for the primary variables in physical units and their interpretations**

**Symbol**	**Interpretation**
*t* ^∗^	Time
*x* ^∗^,*y* ^∗^	Down- and cross-stream positions, respectively
$x^{*}_{\textit {source}}$	Upwind position of source
*Q* ^∗^	Emission rate of detectable puffs
$\bar {U^{*}}$	Constant component of wind speed
*K* ^∗^	Eddy diffusivity
*σ* ^∗^^2^	Plume width parameter
$U^{*}_{\textit {gust}}$	Magnitude parameter for gusts
$r^{*}_{\textit {gust}}$	Mean rate of changes in gust direction
*V* ^∗^	Moth flight speed
$\rho ^{*}_{\textit {puff}}$	Mean number of puffs detected per transit
$r^{*}_{\textit {puff}}$	Detection radius for puffs
$r^{*}_{\textit {source}}$	Detection radius for source

Symbols used in only one section of the analysis are defined in that section and omitted from this table.

**Table 2:** **Summary of the primary rescaled (i.e., non-dimensional) variables in our analyses and their interpretations**

**Symbol**	**Interpretation**
*x*,*y*	Down- and cross-stream positions, respectively
*t*	Time
*x*_*source*_	Upwind position of source
*Q*	Emission rate of detectable puffs
$\bar {U}\equiv 1$	Constant component of wind speed
*K*≡1	Eddy diffusivity
*σ* ^2^≡*x*	Plume width parameter
*U*_*gust*_	Magnitude parameter for gusts
*r*_*gust*_	Mean rate of changes in gust direction
*V*	Moth flight speed
*ρ*_*puff*_	Mean number of puffs detected per transit
*r*_*puff*_	Detection radius for puffs
*r*_*source*_	Detection radius for source

Symbols used in only one section of the analysis are defined in that section and omitted from this table. Scaling is with respect to the length scale, $L = {K^{*} \over \bar {U^{*}}}$ and the time scale $T = {K^{*} \over \bar {U^{*}}^{2}}$. See Section “Simulated plume geometry” for additional details.

An implication of these estimates in the context of Figure 1 is that moths likely confront substantial uncertainty in estimating the lateral positions of pheromone plumes. In both laboratory and field odorant plumes, the probability distribution of puffs in the cross-stream direction occurring at any given streamwise position is often relatively well approximated by a Gaussian distribution (Section “Simulated plume geometry”). The mean of this distribution is an indicator of the cross-stream position of the pheromone source, some distance upstream. Hence, uncertainty about source position is closely associated with uncertainty in the cross-wind location of the corresponding downstream Gaussian distribution of pheromone puffs.

Bayesian estimation using conjugate priors provides an indirect means to assess how moths’ uncertainty may vary across different types of pheromone puff distributions. In Bayesian estimation, the probable centerline position after the *i*th transit is described by a *t*-distribution, with location *μ*_*i*_ and scale $\sigma _{i}/\sqrt {\kappa _{i}}$. Here, *κ*_*i*_ is the effective sample size of pheromone puffs encountered by the end of the *i*th transit, and *μ*_*i*_ and ${\sigma _{i}^{2}}$ are the sample mean and variance of those puffs’ lateral positions (for details, see Section “Conjugate prior analysis for plume geometry” and references therein).

We conjecture that *M. sexta* moths’ processing of spatial information is analogous to Bayesian estimation of the probability distribution of the plume centerline. This conjecture suggests that *κ*_*i*_ is a potentially useful metric of moths’ certainty, and that the lateral extent of cross-plume transits may have an approximately square-root dependence on this metric. Unfortunately, we cannot at present experimentally quantify the locations and times of moths’ in-flight encounters with odorant puffs. Hence, we cannot directly assess whether moths’ movements are statistically related to *κ*_*i*_. Nonetheless, if our conjecture is valid, we predict that (to a useful approximation), the lateral extent of the *i*th transit, *Δy*_*i*_, varies as

(1) $$ \Delta y_{i} = |y_{start_{i}}-y_{end_{i}}|=c^{*}_{0} \kappa_{i}^{-\frac{1}{2}}.  $$

Here, $y_{start_{i}}$ and $y_{end_{i}}$ are the starting and ending lateral positions of the *i*th transit. $c^{*}_{0}$ is a (presently unmeasurable) constant of proportionality that reflects geometrical properties of the plume which are invariant in the laboratory observations.

**Figure 3:**
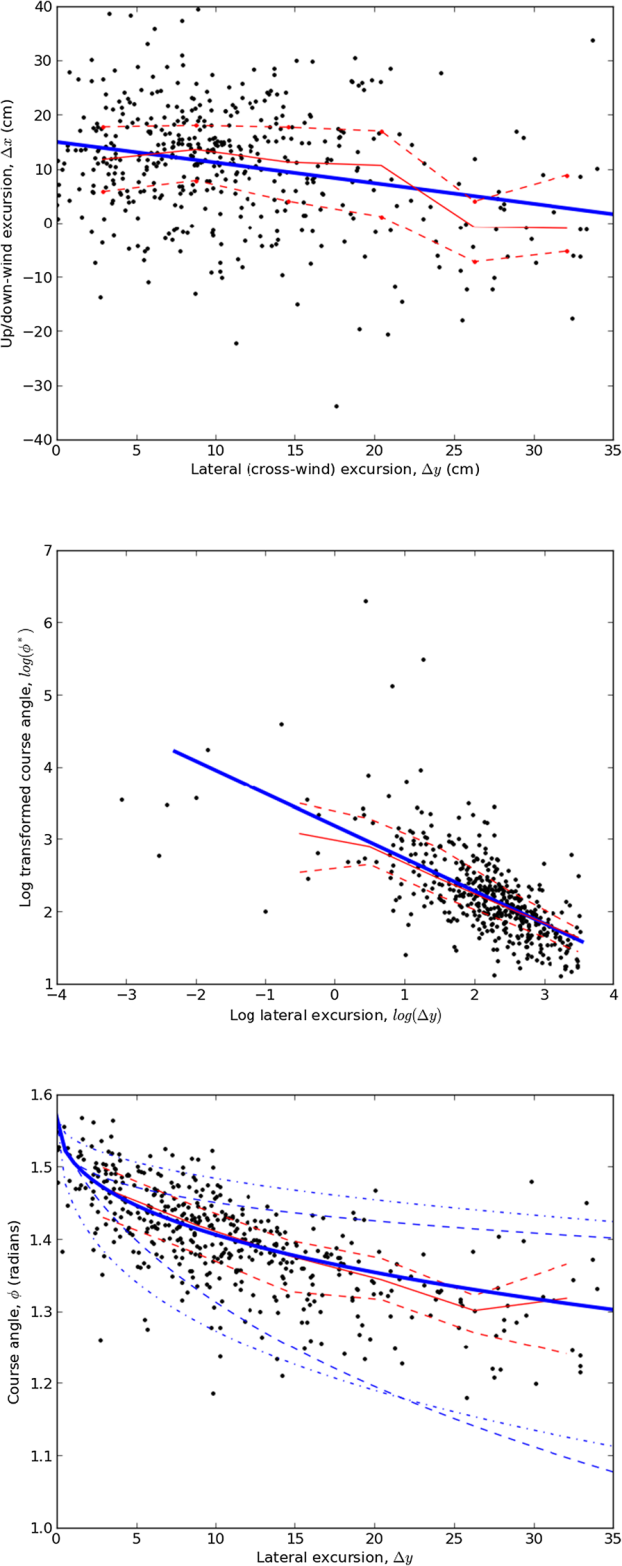
Geometrical characteristics of cross-plume transits in male *M. sexta* moths seeking a pheromone source in a laboratory wind tunnel. (Top) Up/down-stream excursions, *Δ* *x*, plotted against lateral excursions, *Δ* *y*, for all observed transits (*n*=458). A linear regression (blue line, slope = -0.38, intercept = 15.0) is highly significant (*p*≪0.01) but explains little of the variance (*r* ^2^=0.07). (Middle) Log of the transformed course angle, $\phi ^{*}=\frac {\pi }{2|\phi |}-1$, plotted against *l* *o* *g*(*Δ* *y*). The analysis leading to Equation 4 predicts a linear relationship between these quantities. A regression (blue line, slope = -0.45, intercept = 3.18) is highly significant (*p*≪0.01) and explains a substantial part of the variance (*r* ^2^=0.43). (Bottom) Lateral excursion, *Δ* *y*, plotted against course angle, |*ϕ*|. The solid blue curve represents Equation 3 with parameters emerging from the regression, *q*=0.225 and *c*_1_=0.0416. To illustrate effects of parameter variation on this functional form, Equation 3 is plotted with *c*_1_ increased and decreased by a factor of 2 (dot-dashed blue lines) and with *q* increased and decreased by a factor of 1.5 (dashed blue lines). Also shown in all three plots are medians (solid red lines) and 25*th* and 75*th* percentiles (dashed red lines) of binned data.

Cross-stream transits also vary in whether they are upwind or downwind of the previous cross wind track leg (i.e., in up- or down-stream excursion). In the observed trajectories, these excursions varied significantly as a function of *Δy* (Figure 3). Streamwise distances traveled during transits are determined by the angles relative to the wind flown by moths, and by their lateral excursions. We conjecture that a moth’s course angle relative to wind direction depends on its certainty in a way similar to cross-stream distances (i.e., through a power-law dependence on *κ*_*i*_) but with an unknown exponent. We also conjecture, consistent with observations, that course angle varies continuously between nearly perpendicular to the wind when certainty is low (i.e., “casting” when few or no puffs have been recently detected) and almost directly upwind when certainty is high. A simple functional form which conforms to these conjectures is

(2) $$ |\phi_{i}| = \frac{\pi}{2} \frac{1}{1+c^{*}_{1} {\kappa_{i}^{q}}}.  $$

In (2), |*ϕ*_*i*_| is absolute value of the course angle of the *i*th transit measured from the positive *x*-direction. The sign of *ϕ*_*i*_ is determined by the phase of the counter-turning sequence. *q* is an unknown exponent, and $c^{*}_{1}$ is another unmeasurable constant of proportionality.

While (1) and (2) both contain unmeasurable constants, it follows from our conjectures that lateral excursion and course angle are related to each other by

(3) $$ |\phi_{i}| = \frac{\pi}{2} \frac{1}{1+c_{1} \Delta y_{i}^{-2q}}  $$

where $c_{1}=c^{*}_{1} {c_{0}^{*}}^{2q}$ is a constant. With algebraic manipulation, (3) yields

(4) $$ 2 q \log(\Delta y_{i}) - \log(c_{1}) = -\log \left(\frac{\pi}{2 |\phi_{i}|}-1\right)  $$

in which *q* and *c*_1_ can be determined by linear regression over the ensemble of observed transits. The resulting curve-fit (*q*=0.225, *c*_1_=0.0416, *r*^2^=0.43, *p*≪0.01, *n*=458) has encouraging explanatory power over the large variations observed in air course angles and lateral excursions (Figure 3). By comparison, variation in observed airspeeds was relatively small (mean 112 *cm*/*s*, standard deviation 9.9 *cm*/*s*), and a regression of airspeed against lateral excursion (slope=-0.366, intercept=116.5) was significant (*p*≪0.01) but explained little of the variance (*r*^2^=0.072).

Results of this reanalysis suggest two important implications for understanding source-seeking behaviors. First, to a useful approximation, lateral excursion and course angle relative to the wind may be the primary behavioral parameters regulated by source-seeking moths. Second, observed trajectories are consistent with the hypothesis that this regulation reflects puff encounters aggregated via a simple spatial memory.

### Algorithms for spatial memory-based behaviors

The foregoing analysis of observed trajectories of male *M. sexta* moths suggests many potentially feasible and effective algorithms for odor source location based on spatial memory. Here, we present one such algorithm, and simulation results partially characterizing the performance of this algorithm on simplified but nonetheless challenging model pheromone plumes. In this section, we briefly sketch our approach and present simulation results. We provide additional mathematical details and algorithmic variations in Section “Methods”.

In our focal algorithm, we assumed that moths execute a search for a pheromone source by undertaking a sequence of transits across the wind in the horizontal plane (similar to the red lines in Figure 1). This specific behavior has not been observed in any flying moth species but has recently been observed in the desert ant *Catglyphis fortis*. The ants perform this behavior when searching for wind-borne plumes of food odor [50]. In airborne animals like moths, a more mechanistic model of turning aerodynamics would translate this point-to-point translation into a continuously curved trajectory corresponding more closely to observations.

The simulated plume we used to test our algorithm is a modified Gaussian puff model, described in Section “Simulated plume geometry”, in which discrete detectable pheromone puffs with a Gaussian lateral distribution were advected under the influence of a mean wind and episodically shifting lateral gusts. Using the normalization scheme described in that section (see Tables 1 and 2 for a summary of symbols and their interpretations) plumes of arbitrary mean wind speed ($\bar {U}^{*}$) and eddy diffusivity (*K*^∗^) were rescaled to a standard plume $\bar {U}=K=1$. As noted in Section “Simulated plume geometry”, this rescaling has the advantage of reducing the number of parameters. However, a consequence is that typical parameter values differ from their unscaled analogs. For example, in laboratory experiments typical values for emission rates of detectable puffs are often *Q*^∗^≥1*s*^−1^, while the rescaled emission rate $Q=Q^{*}\frac {K^{*}}{\bar {U}^{*2}}$ may (depending on wind speed and turbulence) be substantially lower. Moths detected pheromone puffs only when within detection distance *r*_*puff*_, and located the source only when within detection distance *r*_*source*_. Moths stopped flying when they detected the source.

We assumed that moths maintain a spatial memory in the form of Bayesian conjugate prior hyperparameters, such that at the beginning of each transit they possess updated estimates of the plume’s centerline position and width (Section “Estimates of plume centerline position”), and the density of pheromone puffs within it (Section “Estimates of puff density within the plume”) from previous transits. For simplicity, we assumed that moths decide where a transit will end at the start of that transit, based on information then available (that is, the endpoint of the *i*+1st transit is decided based on puffs encountered during transits *i* and before, but cannot be modified mid-transit). We assumed that moths adjust the lateral extent of each transit to maintain a fixed probability, *P*_*cross*_, that the transit extends past the plume centerline. The cross-stream location at which this probability is achieved is determined by a *t*-distribution specified by Bayesian hyperparameters, as described above and in Section “Estimates of plume centerline position”.

We assumed that moths determine their angle of flight relative to the surrounding air using the functional form (2), with the constants $c^{*}_{1}$ and *q* as behavioral parameters. In particular, this algorithm ensures that low certainty (i.e., low values of *κ*_*i*_) results in casting-like behavior, with movement oriented primarily in the cross-wind direction. Higher certainty (i.e., higher values of *κ*_*i*_) results in movements closer to directly upwind.

If the plume centerline position has moved (e.g., due to gusts or wind shifts), spatial memory of previous puff encounters is likely to be imprecise or downright erroneous as a basis for future movement decisions. In this case, a moth needs to “forget” previous puff encounters and place increased weight on new puff encounters in assessing the new plume position. We assumed that, at the end of each transit, a moth assesses the probability that the plume has moved, *P*_*move*_, by comparing the number of pheromone puffs actually encountered to the previously estimated puff density distribution (see Equation 19, Section “Estimates of puff density within the plume”). We assumed that moths discount hyperparameters associated with previous puff encounters (particularly *κ*_*i*_) in proportion to *P*_*move*_, i.e., by a factor of

(5) $$ 1 - \frac{1}{\tau} P_{move}, ~\tau \ge 1.  $$

Here, *τ* is a timescale parameter that determines the maximum rate at which previous observations are discounted. For example, if *P*_*move*_=1 over a sequence of transits (the extreme case, in which the moth is “certain” that it has lost the plume), previous information about plume characteristics is depreciated and the moth reverts to a substantially uncertain state (i.e., casting behavior) over approximately *τ* transits. We note that if the simulated moth has moved upwind of the source, this algorithm similarly responds to loss of contact with the expected number of pheromone puffs, by decreasing certainty and ultimately reverting to downwind casting movement until the plume is relocated. Real moths that experience decreases in plume encounters due to shifting wind directions or loss of contact with the plume also generate tracks that increase in width with increasing post-plume contact intervals [51,52]. In some species as the post-plume encounter interval increases the moths begin to drift downwind as they increase their track widths [16].

### Simulations of spatial memory-based behaviors

In our simulations, moths’ were assumed to initially encounter the plume during a cross-stream transit. Each individual’s downstream position was drawn from a Gaussian distribution, with mean determined by the pheromone puff nearest a prescribed initial downstream position (here, *x*=500), and standard deviation equal to the puff detection distance, *r*_*puff*_. The lateral starting and ending positions of the initial transit were drawn from a Gaussian distribution with a mean and standard deviation matching the local plume geometry.

**Figure 4:**
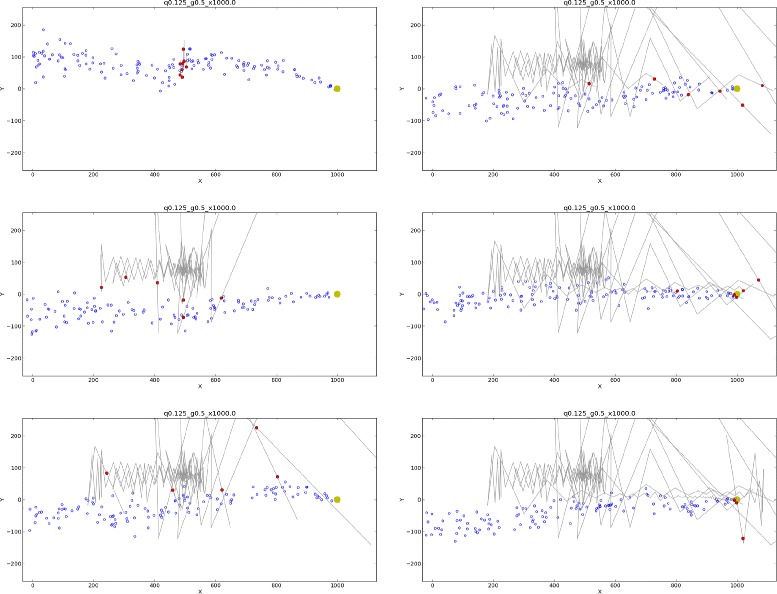
Sample trajectories of simulated male moths seeking a pheromone source. Graphics represent intervals of 250 nondimensional time units, beginning after the initial encounters of 8 moths (*τ*=2.5, *P*_*cross*_=0.25, $c^{*}_{1}=0.8$, *V*=1.15) with a simulated plume (*Q*=0.125, *r*_*gust*_=0.05, *U*_*gust*_=0.5, *r*_*puff*_=*r*_*source*_=10). Red circles represent current moth positions; gray lines represent flight tracks, including the current transit. The plume source is located at *x*=1000, *y*=0. The widely divergent trajectories reflect strong dependence on the location and number of puffs encountered in the initial transit across the pheromone plume, at downstream position *x*=500. In this simulation, the plume of pheromone puffs (blue circles) has been transported by gusts in the positive *y*-direction at the time of encounter. Shortly afterwards, the wind shifts towards the negative *y*-direction, leaving simulated moths well outside the plume. Depending on the level of certainty they attained before the wind shift, moths either advance slowly upwind or begin “casting” while drifting downwind. Upon regaining contact with the plume, moths resume upwind advance. The sequence of images shows several moths “overshooting” the source while undertaking large lateral and upwind excursions; other moths locate and track the plume centerline more closely, and consequently move more directly towards the source. One overshooting moth is shown to revert to casting behavior in response to detecting loss of the plume. At the last time shown, this moth is on a trajectory leading to the source during a sequence of downwind movements.

Typical simulated moth movements within a shifting plume are shown in Figure 4 (also see an animation of simulated movement sequences in Additional file 1). Moth trajectories in our simulations were quite sensitive to stochastic variations in initial encounters with puffs and, when simulated “gusts” were strong, to stochastic variations in plume meander. This is reflected in the substantially different trajectories flown by moths whose randomized initial positions differed only slightly (≤*r*_*puff*_). Similar apparently stochastic differences typically occurred among replicate flights of *M. sexta* moths in the laboratory.

A general feature of moth trajectories in our simulations is that they responded to sharp drops in puff encounters (due to plume shifts, or to moving upstream of the source) in ways qualitatively similar to casting in real moths (that is, by successively widening lateral sweeps, and by decreasing upwind advance and ultimately moving downwind, in an attempt to relocate a lost plume). Simulated moths also responded to increases in puff encounters in ways qualitatively similar to real moths, directing their movements more narrowly upwind. We note that this occurred in our algorithm simply as a consequence of decreased certainty (as quantified in the sample size *κ*_*i*_). That is, there was no behavioral “switch” in the underlying algorithm, but only in the quantitative values of the regulatory hyperparameters associated with spatial memory. Thus, the spatial memory-based behavior we developed here effectively unified these diverse movements along an axis of variation in a single cognitive variable, the uncertainty metric *κ*_*i*_.

#### Searching performance in sparse pheromone puff distributions

To assess searching algorithms, we calculated three performance metrics reflecting distinct aspects of searching effectiveness. We calculated “search success probability”, *P*_*success*_, as the fraction of moths arriving at the source within a fixed (relatively large) time window. *P*_*success*_ primarily reflects accuracy in following a stochastic plume to its source, a small target compared to the spatial extent of the plume and the distance of the downwind starting positions: *P*_*success*_≈1 indicates behaviors that nearly always located the plume (within the time constraint imposed by the duration of the simulation), and *P*_*success*_≪1 indicates behaviors that rarely located the plume. For the subset of moths that were successful, we also calculated two search efficiency metrics: We calculated a time-based efficiency, *E*_*time*_, as the mean straightline flight time from the starting point to the source, divided by the actual time required to locate the source. We calculated a distance-based efficiency, *E*_*distance*_, as the mean straightline distance between moths’ starting position and the source, divided by the actual distance traveled. *E*_*time*_,*E*_*distance*_≈1 indicate behaviors enabling moths to travel in a nearly direct line to the source, while *E*_*time*_,*E*_*distance*_≪1 indicate behaviors causing moths to take highly indirect routes to the source.

**Figure 5:**
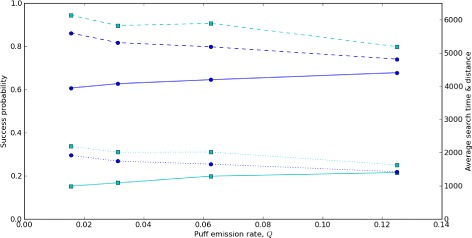
Variation of moth odor source location performance with pheromone puff emmission rate. The lines represent three performance metrics for source location by simulated moths for two different gust rates (blue circles: *r*_*gust*_=0.05; cyan squares: *r*_*gust*_=0.0125) across a range of puff emission rates (*Q*=[0.015625,0.03125,0.0625,0.125]). Lower *r*_*gust*_ corresponds to more persistent gusts, and hence larger lateral plume displacements. Moth behavior parameters are as in Figure 4. Solid lines represent search success probability, *P*_*success*_ – that is, the fraction of moths arriving at the source within a fixed simulated time interval (left axis). Dashed lines represent *E*_*time*_, a time-based efficiency metric for successful moths, i.e., the straightline flight time from the moth’s starting point to the source, divided by the actual travel time (right axis). Dotted lines represent *E*_*distance*_, a distance-based efficiency metric for successful moths, i.e., the straightline distance from the moth’s starting point to the source, divided by the actual distance traveled (right axis). Of the three metrics, only *P*_*success*_ varies strongly between gust conditions; the effects on *E*_*time*_ and *E*_*distance*_ are weaker. Variation across a wide range of puff emission rates has relatively weak effects on all three metrics.

A key motivation in our behavioral analysis was to assess whether a simple spatial memory can confer an ability to track plumes with low signal (sparse pheromone puffs) and high noise (significant turbulence and gust-driven shifts). To quantify sensitivity to signal and noise, we evaluated the three performance metrics to simulated moths in replicated plumes with two gust regimes across a range of puff emission rates (Figure 5). By our assumptions, a hypothetical instantaneous crosswind transit across the full extent of the plume would, on average, result in 2*r*_*puff*_*Q* puff detections (see Equation 7, in Methods). Thus, the metrics in Figure 5 represent variations from a potential average of 2.5 puffs per transit at the highest puff emission rate (*Q*=0.125) to 0.3125 puffs per transit at the lowest puff emission rate (*Q*=0.015625). In these simulations, simulated moths had generally lower success in larger lateral displacements of the plume produced by more persistent gusts (*r*_*gust*_=0.0125) than with smaller lateral displacements produced by more transient gusts (*r*_*gust*_=0.05). However, in both gust regimes, simulated moths maintained remarkably consistent success as reflected by all three performance metrics, even when puff emission rates were so low that almost three quarters of instantaneous cross-plume transits likely resulted in no pheromone puff encounters.

From a physiological perspective, the metrics in Figure 5 also suggest more limited benefits from heightened sensitivity (e.g. threshold changes enabling eight-fold increases or decreases in puff detection rates) than might have been expected. For a pheromone-emitting female, these results also suggest limited benefits to dramatically increased investment in chemical signaling, in terms of the metrics evaluated here. Our results are an interesting counterpoint to the analysis of Andersson et al. [26]. The data they analyzed represent scenarios in which pheromone was emitted at relatively constant rates for long periods relative to source-seeking flight times. Because these insects had a functionally almost unlimited amount of time to locate odorant sources, Andersson et al.’s analysis effectively quantified initial encounter rates of casting insects with pheromone plumes. In contrast, our simulations assumed almost-certain initial contact with at least one pheromone puff, but we imposed time limits on plume duration. Hence our results theoretically assess post-encounter performance, finding that spatial memory-based behaviors substantially reduce impacts of low rates of pheromone puff emission, while Andersson et al. experimentally assessed pre-encounter performance, finding significant impacts of low rates of pheromone puff emission. Additional, complementary studies – theoretical assessments of pre-encounter performance, and experimental assessments of post-encounter performance – are needed to clarify which factors most strongly limit odor source location under natural conditions.

The role played by spatial memory in enabling moths to locate the sources of sparse plumes can be put in context by estimating the effective sample size, *κ*. Assuming the maximum “forgetting” rate corresponding to *P*_*move*_≈1 in (5), the effect of memory over a long sequence of transits is to increase cumulative effective sample size by (minimally) a factor of

$$1 + \left(1 - \frac{1}{\tau}\right) + \left(1 - \frac{1}{\tau}\right)^{2} + \dots=\tau $$

relative to the expected encounter rate for a single transit. For the simulations in Figure 5, in which the memory parameter was *τ*=2.5, effective sample size with spatial memory was at least close to one even for the lowest puff emission rates, and potentially (for transits in which *P*_*move*_≪1) much higher. We note that these are only rough estimates: In our simulations, actual puff encounters varied widely both lower and higher than these estimates (including, e.g., transits that entirely missed the plume, and transits with long upwind excursions through the densest parts of plumes). Nonetheless, these estimates provide useful insight into the degree to which spatial memory might facilitate locating sources of sparse, meandering odorant plumes.

#### Variations in behavioral parameters

**Figure 6:**
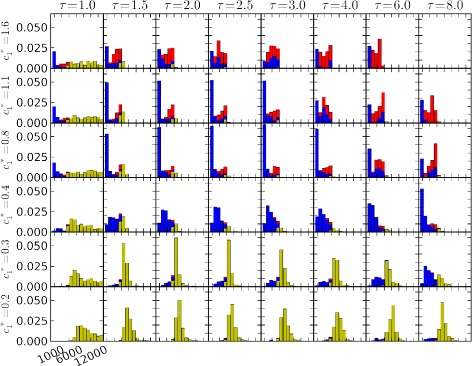
Tabulated outcomes of simulated male moths seeking a pheromone source: variation with memory timescale, ***τ***, and advance parameter, $\boldsymbol {c^{*}_{1}}$. The array of graphs represents a matrix, with columns corresponding to memory timescales *τ*= [1,1.5,2,2.5,3,4,6,8] and rows corresponding to advance parameters $c^{*}_{1}=\,[0.2,0.265,0.4,0.8,1.1,1.6]$. Data comprise 59 replicate plumes (*Q*=0.125, *U*_*gust*_=0.5, *r*_*gust*_=0.05), with 8 replicated moth trajectories in each plume for each combination of *τ* and $c^{*}_{1}$, for a total of 22656 moth trajectories. For all moths, *P*_*cross*_=0.25. Moths were initially 500 nondimensional length units downstream of the source. Simulations were terminated at nondimensional time *t*=12288. In each plot, the horizontal axis represents distances traveled by simulated moths, and the vertical axis represents frequency of trajectories falling into each of 64 bins regularly spaced between 1000 and 12000. Trajectories outside this interval are not shown. Blue indicates moths that successfully found the source; yellow represents unsuccessful moths that “undershot”, i.e., were found downwind of the source at the end of the simulation; red represents unsuccessful moths that “overshot”, i.e., were found upwind of the source at the end of the simulation. These plots suggest combinations of these two parameters that lead to relatively high plume source location probability, centered roughly along a diagonal line from the upper left to near the lower right of the plot matrix. Source location is much less successful both above and below this line, but for different reasons: Above the line, simulated moths are most likely to have overshot the source. Below the line, moths are most likely to have undershot the source.

To assess how effectiveness of the spatial memory algorithm varied with key behavioral parameters, we evaluated our performance metrics across a replicated series of simulations, each with a unique randomly generated plume. In each plume, we simulated a matrix of behavioral parameters, ranging across relevant ranges of *τ* and $c^{*}_{1}$, with 8 replicated moths for each parameter set. Figure 6 graphically summarizes the occurrences of three outcomes in these simulations: “successes”, in which moths flew within detection distance of the source during the simulation; “undershoots”, in which moths failed to find the source and were downwind of it at the end of the simulation; and, “overshoots”, in which moths failed to find the source and were upwind of it at the end of the simulation. In these plots, moths that moved upstream of the source and subsequently returned downstream are counted as undershoots. However, these were infrequent, because moths re-encountering the plume during downstream casting usually advanced upwind relatively quickly, either locating the source or overshooting it again.

In Figure 6, behaviors with the highest success rates occurred in a roughly diagonal band in parameter space, from the upper left (small *τ*, large $c^{*}_{1}$) to the lower right (large *τ*, small $c^{*}_{1}$). Above this band, successes are rarer and most failures are overshoots. Below the band, successes are also rarer but most failures are undershoots. These results lend themselves to intuitive interpretations of the interactions between behavioral parameters *τ* and $c^{*}_{1}$: According to our model, the magnitudes of upwind excursions increase monotonically with $c^{*}_{1}$, and with effective sample size *κ*_*i*_. Increases in *τ* result in larger *κ*_*i*_’s (because puff encounters are “remembered” longer). Hence, for a given set of puff encounters, increased *τ* has broadly the same effect as increased $c^{*}_{1}$ – both result in more rapid upwind advances. If there is a finite range of upwind advance rates within which source location is most effective, we would expect that increases in *τ* can be substantially canceled by reductions in $c^{*}_{1}$, and *vice versa*.

Higher *τ* increases certainty metric *κ*_*i*_ for any given sequence of encounters with pheromone puffs, by extending the longevity of spatial information, while increasing the risk of retaining information that is outdated or misleading due to wind shifts or other stochastic events. Higher $c^{*}_{1}$ increases the upwind advances for any given level of certainty, enhancing the upwind progress extracted from available information, while increasing the risk of missing the source or losing contact with the plume during extended upwind excursions. In this sense, a high- *τ*, high-$c^{*}_{1}$ behavior for locating sources of odorant plumes can be characterized in cognitive terms as a “risk-tolerant” strategy, while a low- *τ*, low-$c^{*}_{1}$ behavior can be characterized as “risk-averse”. Simulation results suggest that both behavioral extremes are likely to be unsuccessful, relative to behaviors with intermediate characteristics.

**Figure 7:**
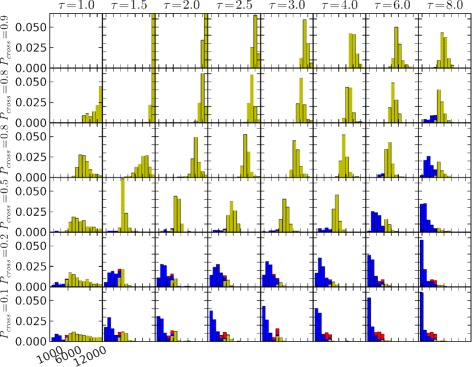
Tabulated outcomes of simulated male moths seeking a pheromone source: variation with memory timescale, ***τ***, and plume-crossing probability, ***P***_***cross***_. The array of graphs represents a matrix, with columns corresponding to memory timescales *τ*= [1,1.5,2,2.5,3,4,6,8] and rows corresponding to plume-crossing probability, *P*_*cross*_= [.125,.25,.5,.75,0.85,0.95]. For all moths, $c^{*}_{1}=0.4$. Data comprise 28 replicate plumes, with 8 replicated moth trajectories in each plume for each combination of *τ* and *P*_*cross*_, for a total of 10752 moth trajectories. Otherwise, interpretation of plots is the same as in Figure 6. These plots suggest combinations of these two parameters that lead to relatively high plume source location probability lie below a diagonal line extending roughly from the lower left to the upper right of the plot matrix. Source location is much less successful above this line, due to the prevalence of “undershoots”. The increasing incident of “overshoots” (red bars) at the bottom right of the plot matrix suggests that the corresponding parameters may be at or near the limits of effective source-seeking behaviors.

We similarly replicated simulations of moth behaviors across relevant ranges of *τ* and *P*_*cross*_ (Figure 7). In this figure, behaviors with the highest success rates occurred in a band just below the lower left to upper right diagonal. Undershoots predominated above this band, with overshoots becoming more frequent below the band in the bottom right corner. Intuitively, larger values of *P*_*cross*_ lead to greater lateral excursions, as transits are required to extend over a greater part of the estimated probability distribution for the plume centerline. Larger values of *τ* contract the estimated centerline probability distribution (by increasing the level of certainty, *κ*), leading to narrower lateral excursions during transits. Lower *P*_*cross*_ decreases the flight time and energy expended on cross-wind movements, while increasing the risk that transits will miss the densest and most informative part of the plume. Hence, in cognitive terms, a high- *τ*, low- *P*_*cross*_ behavior for locating sources of odorant plumes can be characterized as a “risk-tolerant” strategy, while a low- *τ*, high- *P*_*cross*_ behavior can be characterized as “risk-averse”. Behaviors intermediate to these extremes again appear to be the most successful.

#### Performance metrics and “contests”

**Figure 8:**
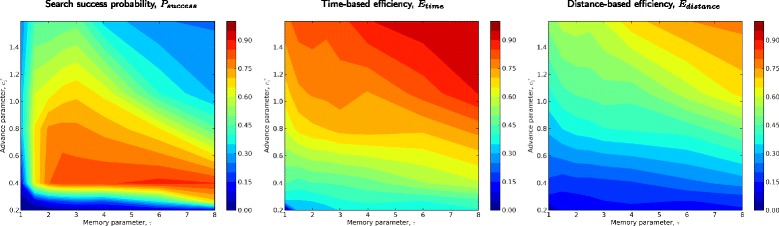
Performance metrics of simulated male moths seeking a pheromone source: variation with memory timescale, ***τ***, and advance parameter, $\boldsymbol {c^{*}_{1}}$. Data and simulation parameters are as in Figure 6. Left: Search success probability, *P*_*success*_; middle: time-based efficiency, *E*_*time*_; right: distance-based efficiency, *E*_*distance*_. Values near 1 (warm colors) indicated good performance; values near 0 (cool colors) indicate poor performance. In all three plots, the horizontal axis represents memory timescale parameter, *τ*, and the vertical axis represents the advance parameter $c^{*}_{1}$. Note the tradeoffs among metrics: The regions of this parameter space maximising success probability corresponds to behaviors with poor efficiency metrics. Conversely, behaviors maximizing the two efficiency metrics (for successful searches) substantially overlap; however, both correspond to regions of relatively low success probabilities.

**Figure 9:**
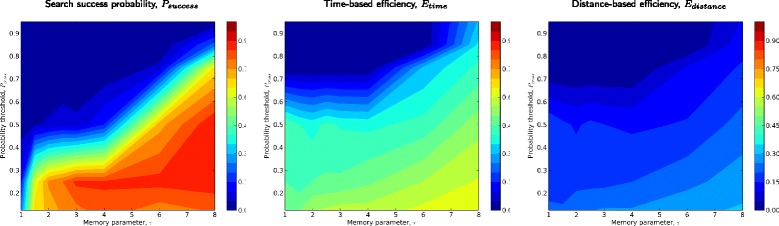
Performance metrics of simulated male moths seeking a pheromone source: variation with memory timescale, ***τ***, and plume-crossing probability, ***P***_***cross***_. Data and simulation parameters are as in Figure 7. Left: Search success probability, *P*_*success*_; middle: time-based efficiency, *E*_*time*_; right: distance-based efficiency, *E*_*distance*_. In all three plots, the horizontal axis represents memory timescale parameter, *τ*, and the vertical axis represents the plume-crossing probability, *P*_*cross*_. Note that success probabilities are maximized by intermediate values of *P*_*success*_. Both efficiency metrics are maximized by lower values of this parameter. Longer memory timescales (higher values of *τ*) broaden the range of *P*_*success*_ corresponding to good source location performance, as indicated by all three metrics.

To gain additional perspectives on odor source location using spatial memory, we evaluated the three performance metrics from Section “Searching performance in sparse pheromone puff distributions” across the *τ*-$c^{*}_{1}$ and *τ*- *P*_*cross*_ behavioral matrices of Section “Variations in behavioral parameters”. In simulations with moderate puff densities (*Q*=0.125) and moderately strong and persistent gusts (*U*_*gust*_=0.5, *r*_*gust*_=0.05), the search success probability performance metric varied from almost always successful (*P*_*success*_≈1) to almost always unsuccessful (*P*_*success*_≈0) over relatively narrow ranges of behavioral parameters (Figures 8, 9). These results suggest that tuning of behavioral parameters, probably with strong dependencies on plume characteristics, is both possible and necessary for algorithms of the type we investigated.

Notably, the shortest memories timescales we simulated (*τ*≈1) had uniformly low *P*_*success*_. In most regions of parameter space, increasing *τ* increased success *P*_*success*_, suggesting longer memory is usually beneficial. This is in line with the general message of our paper. However, there were exceptions, particularly in the upper right of the *τ*-$c^{*}_{1}$ matrix, where overshoots were the dominant mode of failure (Figure 8). In the *τ*- *P*_*cross*_ matrix, increasing *τ* broadens the range of *P*_*cross*_ with high success probabilities. We believe this is because, at higher levels of *τ*, moths’ estimates of the centerline probability distribution become increasingly narrow. Hence, differences in *P*_*cross*_ have relatively weaker effects on transits flown when *τ* is high.

In general, we found close correspondence between time-based and distance-based efficiency metrics. However, parameter combinations with high efficiencies occurred in regions of very low success probability in the *τ*-$c^{*}_{1}$ matrix (Figure 8), and moderately low success probability in the *τ*- *P*_*cross*_ matrix (Figure 9). These plots highlight tradeoffs in source-seeking behavior: Success probabilities are maximized by behaviors with relatively low efficiencies. Conversely, high efficiency behaviors are usually unsuccessful. No single behavior approaches the maximum in both metrics simultaneously.

What is the relationship of metrics like *P*_*success*_, *E*_*time*_, and *E*_*distance*_ to costs and benefits of alternative moth behaviors in a natural, competitive environment? To put the tradeoffs illustrated in Figures 8 and 9 in context, we conducted “contests” in which moths with different behavioral parameters were started within a plume at the same downstream position. The first moth arriving at the source in each contest was the “winner”; if no moth found the source, that contest had no winner. To understand potential consequences of population density, we conducted two-, three- and four-way contests, in which every combination of the corresponding number of moths competed in each replicated plume. We used equal numbers of all parameter combinations represented in Figures 8 and 9. We assessed success by normalizing the number of contests actually won by the number expected if winners had been drawn at random.

**Figure 10:**
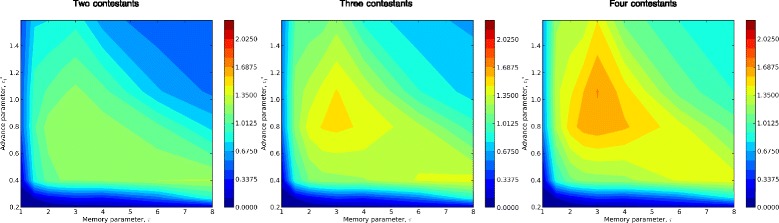
Results of “contests” between simulated male moths seeking a pheromone source: variation across memory timescale, ***τ***, and advance parameter, $\boldsymbol {c^{*}_{1}}$, with fixed plume-crossing probability, ***P***_***success***_ ***=0.25***. In contests, a subset of simulated moths is released within a plume at the same time and downstream position. Analogously to searching by male moths for pheromone-releasing females, the first moth to locate the source “wins”. The plots represent the number of contests won by simulated moths with corresponding parameters, normalized by the expected number if outcomes were random, with two, three or four contestants (left, middle and right, respectively). The overall higher winning metrics for three- and four-way contests reflect the relatively smaller frequency of contests with no winners (i.e., all contestants failed to locate the source). Depending on the number of contestants, two types of odor source location strategies are most successful. One successful strategy corresponds roughly to parameter values ($c^{*}_{1} \approx 0.4$, *τ*≥6) with long search times but nearly maximal search success probability (Figure 8), i.e., to “risk-averse” behaviors. The other successful strategy (roughly $c^{*}_{1} \approx 1$, *τ*≈3) maximizes neither search success probability nor search efficiency, but occupies a region of parameter space in which these metrics are balanced effectively. Because these behaviors fail in locating the source more frequently than risk-averse behaviors, but are faster when they do succeed, they reflect a more “risk-tolerant” strategy. The plots illustrate a shift in contest-winning behaviors away from risk-averse towards risk-tolerant strategies, as the number of contestants increases. This trend reflects strong density-dependence in performance metric tradeoffs: Risk-averse, low efficiency behaviors are likely to win only if there are no successful contestants with risk-tolerant behaviors. As the number of contestants increases, the likelihood that at least one risk-tolerant contestant is successful also increases, shifting the tradeoffs between performance metrics.

**Figure 11:**
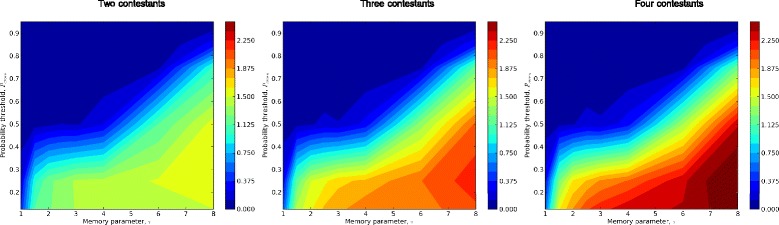
Results of “contests” between simulated male moths seeking a pheromone source: variation across memory timescale, ***τ***, and plume-crossing probability, ***P***_***cross***_, with fixed advance parameter, $\boldsymbol {{c}^{*}_{1}=0{.}9}$. Other details are as in Figure 11. The plots represent the number of contests won by simulated moths with corresponding parameters, normalized by the expected number if outcomes were random, with two, three or four contestants (left, middle and right, respectively). The overall higher winning metrics for three- and four-way contests reflect the relatively smaller frequency of contests with no winners (i.e., all contestants failed to locate the source). These contest outcomes suggest advantages, at least under simulated conditions, for behaviors with longer memory timescales (larger values of *τ*): In two-, three and four-way contests, success increases with memory timescale.

Our contest results suggest restricted ranges of behavioral parameter combinations that were clearly more successful than alternative behaviors (Figures 10, 11). Behaviors that were most successful in contests did not necessarily maximize any of the performance metrics. Instead, contest-winning behavioral strategies generally reflected a balance of the search success probability and search efficiency performance metrics. Different outcomes for contests with different numbers of contestants illustrated what are likely more general density-dependent trends in odor source location performance. One such trend is the overall increase in our contest-winning metric with competitor number. This reflects the lower number of contests with no winner (i.e., in which all entrants failed to find the source) as the number of contestants increased.

A related trend is the apparent shift in contest-winning strategies to higher weighting for efficiency metrics (*E*_*time*_, *E*_*distance*_) relative to searching success metrics (*P*_*success*_) with increasing number of contestants, when these metrics are not maximized in the same regions of parameter space. This is particularly evident in Figure 10, where two disjunct regions of the *τ*-$c^{*}_{1}$ parameter space are relatively successful in three-way contests.

One of these regions represents what might be termed a “slow-but-sure”, risk-averse strategy, corresponding closely to *P*_*success*_-maximizing but relatively inefficient behaviors. The other region represents what might be termed a “hail Mary”, risk-tolerant strategy, significantly shifted towards high efficiency but lower *P*_*success*_ behaviors. At least two interpretations are possible to explain the duality of successful strategies reflected in Figure 10. One is that increasing numbers of contestants generally devalue risk-averse strategies. Another is that risk-averse and risk-tolerant strategies are effectively specializations for plumes that (due to stochasticity in turbulence and gusts) present functionally different characteristics to source-seeking moths. Our simulations suggest that the scope for risk-averse behaviors contracts as the number of contestants increases, but we cannot at present determine whether risk-averse behaviors maintain an advantage even with many contestants for a subset of plume geometries. Both interpretations suggest a diversity of behavioral types, and/or modulation of individual moths’ plume-climbing behaviors as a function of local population density, are likely to be favored under natural conditions. Figure 11 illustrates a complementary case, where (among a different set of behavioral variations) there is broad overlap between performance metrics, and consequently less-pronounced trade-offs between efficiency and search success.

## Conclusions

A comprehensive understanding of odor source location by male *M. sexta* moths and other animals requires validated, quantitative descriptions across a spectrum of organismal levels. At one end of this spectrum are “microscopic” models, describing first-principles biological mechanisms and constraints such as the biomechanics of odorant transport and contact with sensory organs, and the neurophysiology of sensory transduction and motor control. On the other end are “macroscopic” models, describing dynamics at large ecological and evolutionary spatio-temporal scales. In our view, cognitive models of the type developed in this paper, in which behaviors are described in conceptual and statistical terms but are readily identified with microscopic and macroscopic descriptions, occupy intermediate positions on this spectrum, potentially playing useful and necessary roles unifying otherwise disjunct modeling approaches.

Experimental and theoretical studies of mate-seeking behaviors in male *M. sexta* moths, and of analogous behaviors in many other taxa, suggest that odor source location in natural environments is frequently characterized by odorant plumes that are highly patchy and undergo rapid changes. In many, perhaps most, of these cases, encounters with odorant patches are at least intermittently so sparse that information characterizing plume geometry is a potentially important factor limiting searching success. In searching connected with mating, foraging and other key ecological functions, there appear to be strong benefits to “best use” of available information. From a human perspective, “best use” of encounters with plumes suggests statistical approaches involving spatial memory of puff encounters over multiple passes. We conjecture that moths make similar best use, indeed that their counter-turning and casting behaviors are tuned for two equally important objectives: sequentially “sampling” pheromone puff positions, to augment and update spatial memory of plume geometry; and, moving upwind as rapidly as possible while maintaining contact with the plume.

We showed here that implementing effective spatial memory-based searching algorithms for odor source location is theoretically feasible with surprisingly few demands on memory and neural processing. This does not constitute proof that moths employ spatial memory-based searching behaviors, but it does lower the threshold of neural capabilities apparently necessary to make such behaviors possible. We believe the minimal memory and computation requirements of our behavioral scheme substantially erode the *a priori* assumption that such behaviors are impossible within reasonable bounds on moth sensory and information-processing capacities. We also showed that moth movements observed in the lab are consistent with some predictions emerging from our conjectures. In particular, assuming a hidden "uncertainty” metric (the effective sample size, *κ*_*i*_) yielded an interpretation of the relationships among transit geometrical traits that is intuitive and statistically powerful.

The behavior we simulated in this paper represents one of the simplest spatial memory-based searching algorithms. We do not suggest that male *M. sexta* moths use this specific algorithm. Numerous variations of this behavioral theme are easily implemented, some of which are likely more effective or more consistent with observations than the illustrative example presented here. Instead, we emphasize the potential analogies between the neurophysiology of moth sensing and movement; statistical approaches to spatial memory implemented concisely via Bayesian updating and conjugate priors; and, interpretation of behavioral parameters in cognitive terms such as “risk-tolerant” and “risk-averse” strategies. This modeling approach makes it possible to assess, in intuitively and conceptually clear terms, the consequences of changes in hydrodynamic conditions, in females’ chemical signaling behavior, in males’ pheromone detection thresholds, in moth population densities, and in many other factors involved in moths’ odor source location.

For the behavioral variant we simulated, we used performance metrics and “contests” between behavioral variants to assess moth searching success across parameter space. Performance varied strongly over the parameter ranges we investigated. No single set of parameters maximized performance in all metrics. The strong tradeoffs indicated by these results suggest that diverse behaviors may be most successful, at any given instance within the expected spectrum of plume characteristics. Here, our objective was to demonstrate the feasibility of effective source-seeking behaviors with minimal capabilities. We find it plausible, however, that moths and other animals for which source-seeking conveys substantial benefits are endowed with much greater cognitive capabilities, and use those to execute much more flexible, complex and effective search strategies.

## Methods

### Simulated plume geometry

To provide our simulated moths with a geometrically challenging but easily replicated and computationally manageable pheromone distribution, we used a simple 2-dimensional variant of Gaussian plume model. This model mimics transport of discrete, detectable “puffs” of odorant at large time and space scales by an unsteady external flow, and at short time and space scales by correlated random walks that are simplified representations of transport by turbulent eddies. Approximating pheromone distributions as discrete puffs is motivated by fluid mechanical studies of turbulent odorant plumes, which show that odorant remains concentrated in identifiable, discrete blobs and filaments of relatively high concentration (i.e., puffs) far downstream of the source [42,53,54], and that this plume structure is central to source location behaviors [31,32].

Gaussian plumes are widely used to approximate advection due to turbulent environmental flows in both research and applications such as prediction of pollutant distribution (see [55] for a general discussion). These models are typically based on assumptions that downstream transport is dominated by the steady streamwise flow velocity component, and cross-stream transport is dominated by turbulent mixing that can be approximated as acting like a greatly augmented diffusion. From these assumptions it follows that the advected material (female pheromone, in this case) has time-averaged cross-stream concentrations – and, in the case of discrete detectable odorant puffs, probability densities of puff occurrence – that follow Gaussian distributions. Specifically, for odorant puffs dispersed by a constant advection velocity $\bar {U^{*}n}$ in the *x*^∗^-direction and a turbulent eddy diffusivity *K*^∗^, the Gaussian plume model can be expressed as

(6) $$ S^{*}(x^{*},y^{*}) = {Q^{*}/\bar{U^{*}} \over \sqrt{2 \pi {\sigma^{*}}^{2}}} \exp \left[ -{1 \over 2} \left({{y^{*}-\bar{y}^{*}}^{2} \over {\sigma^{*}}^{2}}\right) \right]  $$

where *Q*^∗^ is the rate of puff release (in ${\text {puffs} \over s}$) and ${\sigma ^{*}}^{2}={K^{*} x^{*} \over \bar {U^{*}}}$ is the width parameter (in *m*^2^) of the position *x*^∗^ downstream of the plume source. $\bar {y}^{*}$ is the centerline of the plume in the *y*^∗^-direction [55]. Asterisks denote quantities in physical units; see Table 1 for a summary of these dimensional symbols.

In (6), as in many Gaussian plume models, it is assumed that the total concentration of odorant puffs integrated in the cross-stream direction over the entire lateral extent of the plume is constant in the streamwise direction (that is, there are no downstream sources or sinks of pheromone). We simplify the complex process of pheromone puff detection by assuming that moths detect pheromone puffs if and only if their centers lie instantaneously within a detection radius $r_{\textit {puff}}^{*}$. The expected number of puffs, *ρ*_*puffs*_, detected by a moth transiting rapidly across the plume at downstream position *x* is then approximately

(7) $$ \rho_{puffs}^{*}(x^{*},r_{puff}^{*}) = \int_{x^{*}-r_{puff}^{*}}^{x^{*}+r_{puff}^{*}} \int_{-\infty}^{\infty} S^{*}(x^{*},y^{*}) dy^{*} dx^{*}.  $$

A “nondimensional” form of (6) can be derived by normalizing space and time variables with the length scale $L = {K^{*} \over \bar {U^{*}}}$ and the time scale $T = {K^{*} \over \bar {U^{*}}^{2}}$. This form is given by

(8) $$ S(x,y) = {Q \over \sqrt{2 \pi x}} \exp{\left[ -{1 \over 2} {({y-\bar{y}})^{2} \over x} \right]},  $$

where $x=\frac {x^{*}}{L}=\frac {x^{*} \bar {U^{*}}}{K^{*}}$, $y=\frac {y^{*}}{L}=\frac {y^{*} \bar {U^{*}}}{K^{*}}$ and $t=\frac {t^{*}}{T}=\frac {t^{*} \bar {U^{*}}^{2}}{K^{*}}$ are rescaled space and time variables, and $Q = {K \over U^{2}} Q^{*}$ is the rescaled puff release rate (Table 2). Equation (8) has fewer parameters because the rescaled mean velocity and eddy diffusivity are both unity. In the nondimensional scaling, the expected number of puff encounters for a moth during a rapid lateral transit across the plume is

(9) $$ \rho_{puffs}(x,r_{puff}) = \int_{x-r_{puff}}^{x+r_{puff}} \int_{-\infty}^{\infty} S(x,y) dy~dx.  $$

Equation (8) represents a plume resulting from turbulence in a steady external flow, that does not reflect meander of the plume caused by wind gusts. Meander is a key element of male moths’ odor source location problem. In our Gaussian plume variant, we assume that gusts act only in the cross-stream direction, and are piecewise constant in time and uniform in space. In this case, the centerline position of the plume is $\bar {y}^{*}(t,x)$, a function of time and streamwise position. Consistent with the underlying simplification of the Gaussian plume approach, we neglect unsteady velocity fluctuations in the streamwise direction. To generate a distribution of odorant puffs, we begin with a large ensemble of nondimensional random positions in the horizontal plane, *x*_*i*_,*y*_*i*_,*i*=1,2,…,*N*_*puffs*_. Here, *y*_*i*_ is chosen from a standard normal distribution, and *x*_*i*_ is chosen from a sorted list of samples from a uniform distribution on the interval [0,1]. *N*_*puffs*_ is an ensemble size large enough to exceed the number of puffs leaving the source during the source-seeking simulation. A distribution of nondimensional gust arrival times *t*_*j*_,*j*=1,2,…,*N*_*gusts*_ is similarly chosen from a sorted list of samples from a uniform distribution on the interval [0,1]. Corresponding nondimensional gust magnitudes *v*_*i*_ (that is, gust magnitudes rescaled by *L* and *T*) are chosen from a semicircular distribution with unit radius.

To implement gust-induced meander in our plume model, we rescale these random variables so as to mimic key characteristics of odorant puffs released at a fixed source location at random time intervals, and advected by a spatially uniform wind field whose direction is episodically modified by changes in gust direction. Rescaled puff release times are given by $\tilde {t}_{j}=\frac {N_{\textit {gusts}}}{r_{\textit {gust}}} t_{j} - x_{\textit {source}}$. Rescaled streamwise puff positions are given by $\tilde {x}_{i}=\frac {N_{\textit {puffs}}}{Q} x_{i}$. At time *t*, only puffs for which $\tilde {x}_{i} < x_{\textit {source}}+t$ have emerged from the source; the streamwise positions of those puffs is given by $X_{i} = \tilde {x}_{i} - t$. Rescaled cross-stream positions are given by $\tilde {y}_{i}=\sqrt {x_{\textit {source}}-X_{i}}~y_{i}$. Rescaled puff magnitudes are given by *V*_*j*_=*W*_*gust*_*v*_*j*_. The cross-stream positions of emerged puffs are $Y_{i} = \tilde {y}_{i} + y_{\textit {disp}}(t)$, where $y_{\textit {disp}}(t)=\bar {y}(t)-\bar {y}(t-x_{\textit {source}}+X_{i})$. $\bar {y}(t)$ is linearly interpolated from $y_{\textit {disp}}(\tilde {t}_{k})=\sum _{j<k} V_{j+1}(\tilde {t}_{j+1}-\tilde {t}_{j}),~k=1, 2, \dots,N_{\textit {gusts}}$.

This plume model does not explicitly represent fluid dynamics. However, it does incorporate the type of short- and long-term fluctuations in fluid flow that make the source-location problem difficult. Because it is extremely quick to simulate, and because the data files required to fully reconstruct a plume realization are small, this plume model makes a good test-bed on which to assess behavioral algorithms. In particular, this algorithm makes it easy to explicitly modulate intermittency and cross-stream meander, the key characteristics that make plume-following challenging to organisms under real-world conditions.

### Conjugate prior analysis for plume geometry

#### Estimates of plume centerline position

To model moths’ estimation of plume geometry, we drew on standard Bayesian theory [47] by adopting a conjugate prior probability density for mean *μ* and variance *σ*^2^ of puff position in the Gaussian puff distribution after the *i*th transit,

(10) $$ \begin{aligned} P\{\mu, \sigma^{2} \}_{i} &= N Inv \chi^{2}\left(\mu_{i},{\sigma^{2}_{i}}/ \kappa_{i};\nu_{i},{\sigma^{2}_{i}}\right)\\ &\quad\propto N\left(\mu_{i},{\sigma^{2}_{i}}/ \kappa_{i}\right) Inv\chi^{2}\left(\nu_{i},{\sigma^{2}_{i}}\right). \end{aligned}  $$

In (10), $Inv\chi ^{2}(\nu _{i},{\sigma ^{2}_{i}})$ is an Inverse- *χ*^2^ distribution with degrees of freedom *ν*_*i*_ and scale *σ*_*i*_, representing the marginal probability density of the variance, *σ*^2^, after the *i*th transit. $N(\mu _{i},{\sigma ^{2}_{i}}/ \kappa _{i})$ is a normal distribution with mean *μ*_*i*_ and variance ${\sigma ^{2}_{i}}/ \kappa _{i}$, representing the probability density of the plume centerline, *μ*, conditional on variance *σ*^2^, after the *i*th transit. After the *i*+1st transit in which *n*_*i*+1_ puffs were detected at lateral positions *y*_*i*_,*i*=1,2,…,*n*_*i*+1_ with sample mean $\bar {y}_{i+1}$ and sample variance $s^{2}_{i+1}$, the updated posterior distribution is

(11) $$ P\{\mu, \sigma^{2} \}_{i+1} = N Inv \chi^{2}\left(\mu_{i+1},\sigma^{2}_{i+1}/ \kappa_{i+1},\nu_{i+1},\sigma^{2}_{i+1}\right),  $$

where

(12) $$ {\small{\begin{aligned} {} \mu_{i+1} =\frac{\kappa_{i} \mu_{i}+n_{i+1} \bar{y}_{i+1}}{\kappa_{i}+n_{i+1}}, \kappa_{i+1}=\kappa_{i}+n_{i+1}, \nu_{i+1}=\nu_{i}+n_{i+1}, \\ {} \nu_{i+1} \sigma^{2}_{i+1}=\nu_{i} {\sigma^{2}_{i}}+(n_{i+1}-1)s^{2}_{i+1}+\frac{\kappa_{i} n_{i+1} }{\kappa_{i}+n_{i+1}} (\bar{y}_{i+1}-\mu_{i})^{2}. \end{aligned}}}  $$

The marginal distribution of plume centerline $\bar {y}_{i+1}$ given the posterior distribution after the *i*+1st transit is then,

(13) $$ P\{\bar{y}_{i+1}\}=t_{\nu_{i}}(\bar{y}_{i+1}| \mu_{i},{\sigma^{2}_{i}}/\kappa_{i}),  $$

i.e., a *t*-distribution with degrees of freedom *ν*_*i*+1_, location *μ*_*i*+1_, and scale $\sigma _{i+1}/\sqrt {\kappa _{i+1}}$.

We assume moths modulate lateral excursions such that transits cross the plume centerline with probability *P*_*cross*_. This is achieved by selecting the endpoint of the *i*+1st transit to be equal to the more distant limit of the central posterior interval, *CPI*, of the distribution in (13),

(14) $$ y_{end_{i+1}} = CPI(P\{\bar{y}_{i}\},P_{cross})- y_{end_{i}},  $$

i.e., the limit that maximizes $|CPI(P\{\bar {y}_{i}\},P_{\textit {cross}})- y_{end_{i}}|$. Here, the ending location of the *i*th transit is the starting location of the *i*+1st transit, $y_{start_{i+1}}=y_{end_{i}}$.

#### Estimates of puff density within the plume

To model moths’ estimation of plume density, we assume puff encounters are Poisson-distributed random events with mean *ρ*_*puff*_, such that the probability of encountering *k* puffs on a given transit is

(15) $$ P\{k|\rho_{puff}\}=\frac{\rho_{puff}^{k} e^{-\rho_{puff}}}{k!}~.  $$

The conjugate prior probability density for plume density, *ρ*_*puffs*_, is

(16) $$ P\{\rho_{puff} \}_{i} = Gamma(\alpha_{i}, \beta_{i}).  $$

Here, *α*_*i*_ corresponds to the cumulative number of puffs detected, *β*_*i*_ to the cumulative number of transits, and *α*_*i*_/*β*_*i*_ to the mean of the prior distribution for *ρ*_*puffs*_. After the *i*+1st transit, the updated posterior distribution is

(17) $$ \begin{aligned} P\{\rho_{puff} \}_{i+1} &= Gamma(\alpha_{i+1}, \beta_{i+1}),\\ \alpha_{i+1} &= \alpha_{i}+n_{i+1}, \beta_{i+1}=\beta_{i}+1. \end{aligned}  $$

The marginal probability of puffs encountered in the *i*th transit, *k*_*i*_, is a negative binomial distribution,

(18) $$ P\{k_{i}\} = Neg-bin(\alpha_{i},\beta_{i}).  $$

The probability that, given the history of puff encounters encoded in the hyperparameters *α*_*i*_ and *β*_*i*_, *k*_*i*_ or fewer puffs are encountered in a given transit is the Cumulative Distribution Function of (18), *CDF*. Here, we assume that moths use the *CFD* to quantify the probability, *P*_*move*_, that the plume has shifted due to wind gusts (or, equivalently, that the moth has lost the plume due to overshooting or poor movement choices),

(19) $$ P_{move}=CDF(Neg-bin(\alpha_{i},\beta_{i}),k_{i}).  $$

#### Detecting wind shifts and overshoots

The Bayesian conjugate prior hyperparameters associated with plume geometry and puff density are derived assuming that the distributions being estimated are statistically stationary. The increased precision with which stationary plume characteristics can be estimated as successive puffs are encountered are reflected by the hyperparameters *κ*_*i*_, *ν*_*i*_ and *β*_*i*_. As these hyperparameters get larger, the corresponding distributions (10) and (18) become increasingly constrained, while the marginal effects of additional samples become smaller.

Under field conditions, transients such as gusts and wind-shifts often cause pheromone plumes to undergo significant changes in geometry during males’ searches for females. In addition, moths can “overshoot” the source, or be misled by incomplete sampling of the puff distribution or by stochastic variations in puff encounters. In these cases, moths need a mechanism to decrease their reliance on previous puff encounters, and increase their responsiveness to new puff encounters. This corresponds to discounting the effective sample size hyperparameters, through multiplication with a discounting factor, 0<*f*≤1. To insure that this discounting lowers certainty without altering current estimates of plume geometry and density, the cumulative variance, $\nu _{i} {\sigma ^{2}_{i}}$, and total puffs encountered, *α*_*i*_, must be similarly discounted. Hence, “forgetting” of obsolete or erroneous estimates of plume density are made possible by a modification of (17), such that

(20) $$ \alpha_{i}=f \alpha_{i} + n_{i+1}, \beta_{i}=f \beta_{i} +1,  $$

and of (12), such that

(21) $$ \begin{aligned} {}\kappa_{i+1}&=f \kappa_{i}+n_{i+1}, \nu_{i+1}=f \nu_{i}+n_{i+1}, \\ {}\nu_{i+1} \sigma^{2}_{i+1}&=f (\nu_{i} {\sigma^{2}_{i}})\,+\,(n_{i+1}\,-\,1)s^{2}_{i+1}\,+\,\frac{\kappa_{i} n_{i+1}}{\kappa_{i}\,+\,n_{i+1}} (\bar{y}_{i+1}\,-\,\mu_{i})^{2}. \end{aligned}  $$

A number of schemes are plausible to determine the discounting factor. For example, an algorithm in which the memory is discounted as a function of elapsed time is

$$ f = e^{-\delta t/\tau}, $$

where *Δt* is the duration of the previous transit and *τ* is a memory timescale. Discounting of this form was used by [56] to model spatial memory and cognition in schooling fish.

An algorithm in which the memory is discounted as a function of transit number is given by

$$f = 1-\frac{1}{\tau}. $$

Here, we assume that moths use (19) to assess the probability that the plume has moved (or that they have lost it), adjusting the discount factor as

(22) $$ f = 1-\frac{1}{\tau} P_{move}.  $$

With this algorithm, discounting is strongest when indications are present that the plume has moved (*P*_*move*_≈1), while discounting is almost entirely absent when no such indications are present.

## Additional file

Additional file 1
**Moth simulation animations.** An animation of the simulated moth trajectories in Figure 4 can be viewed at this URL: http://faculty.washington.edu/random/movie2_g0.5_q0.125.avi.
